# Retropharyngeal, Parapharyngeal and Peritonsillar Abscesses

**DOI:** 10.3390/children9050618

**Published:** 2022-04-26

**Authors:** Susanna Esposito, Claudia De Guido, Marco Pappalardo, Serena Laudisio, Giuseppe Meccariello, Gaia Capoferri, Sofia Rahman, Claudio Vicini, Nicola Principi

**Affiliations:** 1 Pediatric Clinic, Pietro Barilla Children’s Hospital, Department of Medicine and Surgery, University of Parma, Via Gramsci 14, 43126 Parma, Italy; claudia.deguido@studenti.unipr.it (C.D.G.); marco.pappalardo@studenti.unipr.it (M.P.); serenalaudisio@gmail.com (S.L.); gaia.capoferri@unipr.it (G.C.); sofia.rahman@unipr.it (S.R.); 2 Head-Neck and Oral Surgery Unit, Department of Head-Neck Surgery, Otolaryngology, Morgagni Piertoni Hospital, 47121 Forlì, Italy; otorino.fo@auslromagna.it (G.M.); claudio@claudiovicini.com (C.V.); 3 Università degli Studi di Milano, 20122 Milan, Italy; nicola.principi@unimi.it

**Keywords:** deep neck infections, parapharyngeal abscess, peritonsillar abscess, retropharyngeal abscess

## Abstract

Deep neck infections (DNIs) include all the infections sited in the potential spaces and fascial planes of the neck within the limits of the deep layer of the cervical fascia. Parapharyngeal and retropharyngeal infections leading to parapharyngeal abscess (PPA) and retropharyngeal abscess (RPA) are the most common. DNIs remain an important health problem, especially in children. The aim of this narrative review is to describe the management of peritonsillar, retropharyngeal and parapharyngeal abscesses in pediatric age. Despite relatively uncommon, pediatric DNIs deserve particular attention as they can have a very severe course and lead to hospitalization, admission to the intensive care unit and, although very rarely, death. They generally follow a mild upper respiratory infection and can initially present with signs and symptoms that could be underestimated. A definite diagnosis can be made using imaging techniques. Pus collection from the site of infection, when possible, is strongly recommended for definition of diseases etiology. Blood tests that measure the inflammatory response of the patient may contribute to monitor disease evolution. The therapeutic approach should be targeted toward the individual patient. Regardless of the surgical treatment, antibiotics are critical for pediatric DNI prognosis. The diagnostic-therapeutic procedure to be followed in the individual patient is not universally shared because it has not been established which is the most valid radiological approach and which are the criteria to be followed for the differentiation of cases to be treated only with antibiotics and those in which surgery is mandatory. Further studies are needed to ensure the best possible care for all children with DNIs, especially in this era of increased antimicrobial resistance.

## 1. Background

Deep neck infections (DNIs) include all the infections sited in the potential spaces and fascial planes of the neck within the limits of the deep layer of the cervical fascia. Parapharyngeal and retropharyngeal infections leading to parapharyngeal abscess (PPA) and retropharyngeal abscess (RPA) are the most common [1,2]. Moreover, as the peritonsillar space cannot be considered among the deep spaces of the neck, peritonsillar abscesses (PTA) are usually considered a component of the DNI group [1,2]. In the past, several cases of DNIs had a very poor prognosis, because of the late diagnosis, the higher incidence of complications and the poor efficacy of medical and surgical therapies [3]. More recently, advances in diagnostic imaging, the availability of broad-spectrum antibiotics capable of overcoming the emerging bacterial resistance and the refinement of the surgical procedures have made diagnosis easier and earlier and therapy significantly more effective. Despite this, DNIs remain an important health problem in both children and adults.

Precise localization of the infection remains difficult due the complex anatomy of the deep neck spaces and the risk that the infection site is covered by a substantial amount of unaffected superficial soft tissue. Surgical access to the site of infection requires crossing of superficial tissues with the risk of injury of the underlying neurovascular and soft tissue structures [4,5]. Early identification of suspected DNIs, a rational use of the available diagnostic measures, the use of the most appropriate antibiotic therapy and the right selection of patients for whom a surgical approach is needed remain essential to assure a rapid resolution of the infection and a good short- and long-term prognosis. Children deserve special attention as in several cases achieving these goals can be very difficult. Early diagnosis may be delayed as initial symptoms are insidious as they overlap with those of common upper respiratory infections and the poor verbal communication may make it difficult to report signs and symptoms of the disease [4,5]. Moreover, due to the different neck structure, mainly the fact that lymph nodes are more predominant in different anatomical sites, children have different clinical manifestations and a different disease etiology than adults [6,7,8]. Knowledge of these differences is critical for an effective approach to children with DNIs. The aim of this narrative review is to describe the management of peritonsillar, retropharyngeal and parapharyngeal abscesses in children. A literature search was performed using the PubMed database, with a selection of English-language articles published from 2001 to 2021. The articles were selected using the key words: “children” or “pediatric” or “paediatric” and “deep neck infection” or “retropharyngeal abscess” or “parapharyngeal abscess” or “peritonsillar abscess”.

## 2. Neck Anatomy and Deep Infection Development

**Figure 1:**
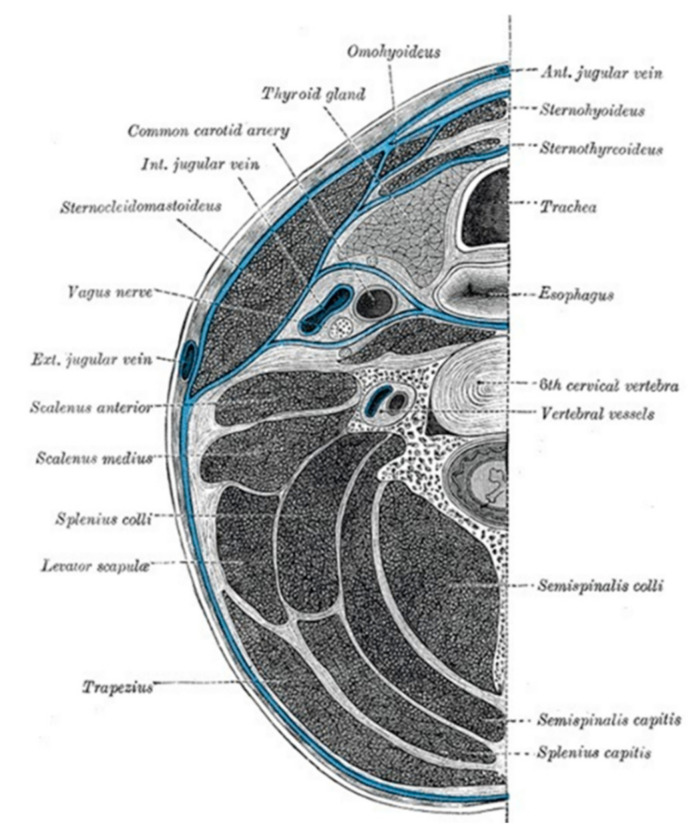
Deep cervical fascia of the neck (blue color), being well-demonstrated by this transverse section at the level of the sixth cervical vertebra. Licensed under CC by 4.0. Available from: https://www.ncbi.nlm.nih.gov/books?term=transverse%20section%20of%20the%20neck%20AND%20book_statpearls[sb]&report=imagesdocsum (Accessed on 18 March 2022).

The head and neck are completely enveloped by fascial layers that support internal structures and help to compartmentalize neck components [9]. Two cervical fascias exist. The superficial and the deep fascias. The superficial cervical fascia lies between the dermis and the deep cervical fascia and does not constitute part of the deep neck space system. The deep cervical fascia is divided into superficial, middle and deep layers (Figure 1).

**Figure 2:**
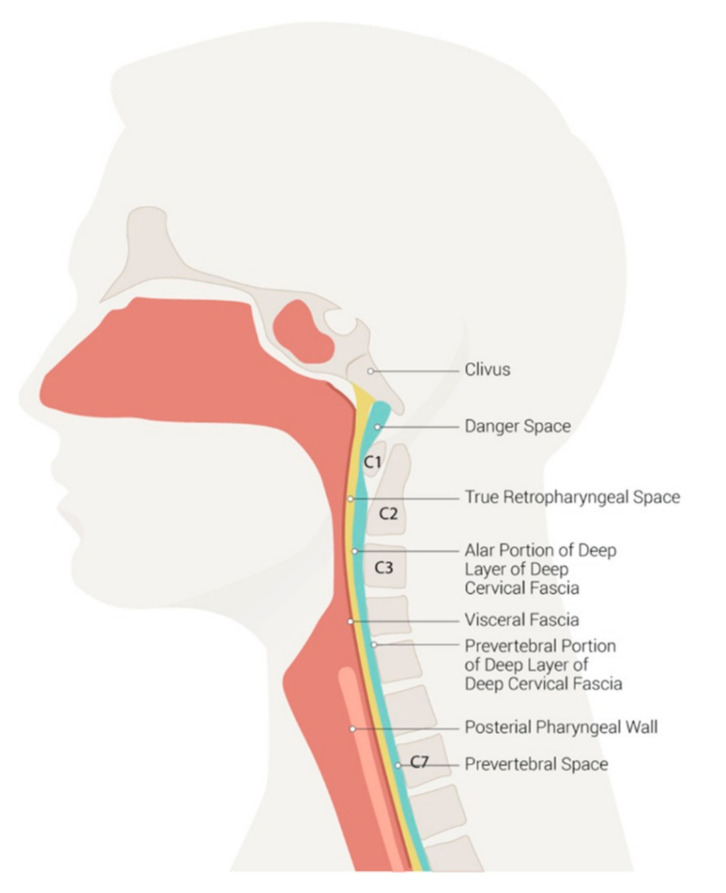
Neck spaces. Licensed under CC by 4.0. Available from: https://www.ncbi.nlm.nih.gov/books/NBK537044/figure/article-36304.image.f1/ (Accessed on 18 March 2022).

All the 11 spaces that are created by planes of greater and lesser resistance between the fascial layers can be the site of infection. Organs and related lymph nodes are covered by the single layer condition of the site that is infected. The superficial layer of the deep cervical fascia, also named the investing layer, covers the submaxillary and parotid glands, and the trapezius, sternocleidomastoid and strap muscles, and enclose two spaces, the masticator space and the suprasternal space [9]. Infections of odontogenic and submandibular origin affect these spaces. The middle layer encloses the visceral space and parapharyngeal space. They contain the buccinator muscle, pharynx and pharyngeal constrictor muscles (superior, middle and inferior), cervical esophagus, thyroid and parathyroid glands, trachea, larynx, visceral lymph nodes and recurrent laryngeal nerve. The space deep in the visceral layer of the deep cervical fascia encircling the pharyngeal constrictor muscles between the skull base and cricoid cartilage is named by some authors as the pharyngeal mucosal space; within it, nasopharyngeal, oropharyngeal and hypopharyngeal divisions are reported [10,11]. Infections of pharyngeal, tonsillar and laryngeal origin together with infections of the 2nd and 3rd molars can involve these spaces, more frequently causing PPA. Moreover, as the parapharyngeal space can communicate with the brain through various foramina, infection can extend to the nervous system [12]. The deep layer of the deep cervical fascia, also called prevertebral fascia, encases the paravertebral muscles, and forms the perivertebral space. It consists of the perivertebral fascia and alar fascia [13]. The space between the alar fascia and the prevertebral fascia is the danger space as it is in continuity with the mediastinum and infections of upper aerodigestive origin can spread freely to lung structures. The space between the alar fascia and the posterior aspect of the middle layer of the deep cervical fascia is the retropharyngeal space. The retropharyngeal space extends longitudinally from the base of the skull to the mediastinum, at the level of the second thoracic vertebra. Figure 2 shows the neck spaces.

The lymph nodes that drain the adenoids, sinuses, nose and pharynx are in this space. Infections in any of these areas can result in spread of infection to these lymph nodes, resulting in lymphadenitis and RPA development [14]. PTA is a localized infection where pus accumulates between the fibrous capsule of the tonsil and the superior pharyngeal constrictor muscle [15]. Pathogens spread to the peritonsillar space via the salivary system after an oral or a pharyngeal bacterial infection [16].

## 3. Epidemiology, Etiology and Clinical Manifestations

### 3.1. Epidemiology

After the introduction of broad-spectrum antibiotics in clinical practice, incidence rates of DNIs in children significantly decreased. Similarly, the complications and mortality rates of the remaining cases, which were 25% in the pre-antibiotic era, declined significantly, despite less than the absolute incidence rate [17]. However, starting from the beginning of this century, an increase in hospital admissions of pediatric DNIs has been reported by some centers, leading to a marked increase in the total inflation-corrected hospital charges for these diseases [18,19]. However, more in-depth analyzes seem to indicate that the increase did not regard all the DNI types and that the increase in the number of hospitalized cases occurred only where RPAs were the most diagnosed DNIs. A national study carried out in the USA has shown that in the period 2000–2009 the incidence of pediatric RPA was significantly raised from 0.10 cases per 10,000 to 0.22 in 2009 (*p* = 0.02) [20]. On the contrary, despite being slightly increased, the incidence rates of the combined DNIs (1.07–1.37 cases per 10,000, *p* = 0.07), PTA (0.82–0.94 cases per 10,000, *p* = 0.12) or PPA (0.08–0.14 cases per 10,000, *p* = 0.13) were not significantly modified [18]. The emergence of drug-resistant bacterial strains, mainly methicillin-resistant Staphylococcus aureus (MRSA), in normal oropharyngeal flora is considered the most important cause of this phenomenon [21]. The evidence that, in a cross-sectional retrospective analysis of pediatric DNIs visited in a Portuguese hospital from 2011 to 2016, most children had previously received ineffective (although theoretically rational) antibiotic treatment, supports this conclusion [22].

Despite DNIs being able occur in all pediatric ages, they are more common among children younger than 5 years of age, and among them RPA and PPA predominate. On the contrary, PTAs are more frequently diagnosed among adolescents [18,23,24]. Higher incidence rates of RPAs and PPAs in preschool children depends on both infectious and anatomical factors. In the first years of life, upper respiratory tract infections and cervical adenitis are significantly more common. Moreover, in the same period of life the paramedian chain of lymph nodes in the retropharyngeal space is prominent, whereas it tends to involute after the fifth years of age. RPAs and PPAs are typically caused by the suppuration and perforation of lymph nodes, which serve as a drainage pathway of body sections of the upper respiratory tract [25,26]. PTAs generally occur in older children and adolescents as complication of an initial acute bacterial pharyngotonsillitis, a disease that is more common in this age groups [27]

### 3.2. Etiology

Several bacterial pathogens are considered potential etiologic agents of DNIs. In most of the studies the most commonly detected pathogens were *Streptococcus* species (α-hemolytic and β-hemolitic streptococci and *Streptococcus anginosus* group) and *Staphylococcus aureus*, detected alone or in combination with each other or with other pathogens, both aerobes and anaerobes. Among anaerobes, *Fusobacterium, Peptostreptococcus* and *Porphyromonas* are the commonest [18,28,29,30,31], although the role of these bacteria as etiologic agents of DNIs is debated. The evidence that in children with acute pharyngotonsillitis, conditions that can precede DNI development, detection of *Fusobacterium necrophorum* was more frequently detected than in healthy children [29], seems to suggest that anaerobes can have a potential pathogenetic role. Further support to this hypothesis is given by the report that in children with PTA mixed flora with anaerobes is more common than the aerobic flora alone [32]. Finally, very few cases due to Mycobacterium tuberculosis have been reported [33,34].

However, the frequency of detection of the pathogens differs in children compared to adults and in younger children compared to adolescents. The etiology is strictly dependent on the site of the infection from which pathogens causing DNI derive. As already reported, most pediatric DNIs develop from an upper respiratory tract infection with suppurative lymphadenitis and are consequently due to one or more of the microorganisms that usually colonize the upper respiratory tract and cause these diseases. Adolescent and adult cases, on the contrary, are mainly associated with streptococcal pharyngitis and a dental pathology that are generally caused by different organisms. A series of studies have clearly evidenced these differences [7,8,35,36,37]. A good example in this regard is given by the study by Shimizu et al. [7], who compared the pus culture of 15 children and 99 adults. In children the most common pathogens were *Staphylococcus* and *Streptococcus* species (60% and 27%, respectively), whereas *Streptococcus anginosus* and anaerobes were identified in only one and two cases. In adults, *Staphylococcus aureus* was identified in less than 10% of cases. In addition, the role of *Streptococcus* species and anaerobes were significantly greater (61% and 46%, respectively) [7].

### 3.3. Clinical Manifestations

Children with DNI often present with a prodromal illness with upper respiratory tract symptoms with or without fever [7,8]. Progression to signs and symptoms evidencing the neck structures’ involvement is generally rapid. Clinical manifestations of a DNI can differ according to age and infection location. Subjective complaints, such as sore throats, voice changes and odynophagia, can be difficult or impossible to evidence in infants and young toddlers [7,8]. This explains why in the first months of life DNIs can be initially considered as upper respiratory tract infections only, the involvement of the deep structures is not taken into account properly and the disease is strongly suspected only when a septic picture is already developed. Regarding infection location, it must be highlighted that all the DNIs are characterized by common signs and symptoms such neck swelling and pain, limited neck motion, trismus and reduced oral intake in most of the cases associated with fever and cervical lymphadenopathy. Moreover, for each deep neck space specific clinical manifestations can develop according to the neck structures that are within or near the space [32]. However, in uncomplicated cases, only peritonsillar infections, especially when a PTA develops, can be diagnosed on the base of clinical findings alone. In this case diagnosis can be strongly suspected on the base of severe unilateral sore throat, cervical lymphadenopathy, tonsillar or pharyngeal exudates, uvular deviation toward the unaffected side and upper airway obstruction [32]. In all the other DNIs, identification of the site of the infection only on the base of clinical manifestations is significantly more difficult mainly because infection can rapidly spread within communicating spaces and different organs sited in different spaces can be simultaneously affected [32]. Only when complications involving specific organs occur is it easier to identify the initial site of the infection. If there is a parapharyngeal space infection, swelling in the submandibular triangle and medial displacement of the lateral pharyngeal wall are evidenced. Moreover, risk of respiratory and vascular problems is significant, as in this space the cranial nerves VIII, X and XII, carotid heaths, and cervical sympathetic trunk are situated. Internal jugular vein thrombosis (Lemierre syndrome), internal carotid artery erosion and laryngeal edema can develop [32]. Finally, the presence of large abscesses and sudden-onset laryngeal edema can lead to acute airway obstruction [32]. Retropharyngeal space infections cause general signs and symptoms quite similar to those detected in parapharyngeal space infections. Risk of respiratory failure is even greater. Moreover, infections can spread to the chest and to the prevertebral space, leading to very severe complications in these areas. Lungs can be involved in the form of pleural empyema and the cardiac system can be affected, with patients developing pericarditis, pericardial effusion [24,38,39,40,41,42].

Studies carried out in children seem to indicate that, in most cases, pediatric DNIs are diagnosed before severe complications arise. In a study enrolling 25 children with a mean age of 5.6 months, hospital admission occurred about 4 days after disease onset [43]. A neck mass or swelling was evidenced in 95% of the cases and cervical lymphadenopathy in 67%. Fever was detected in 60% and poor food intake in 36% [43]. Similar findings were reported in a recent evaluation of 159 hospitalized patients, among whom 102 were school-age children (mean age of 4.4 years) and 57 were adolescents (mean age 13.8 years) [25]. It was confirmed that the initial clinical manifestations of pediatric DNIs can simulate a common upper respiratory tract infection with fever documented in 63.9% of cases, odynophagia in 50.6%, pharyngeal bulging in 46.1% and neck mass in 35%. Interestingly, it was reported that pharyngeal bulging with uvular deviation and trismus may alert to a PTA, whereas these signs may not be so obvious in PPA and RPA [32]. Moreover, fever was the only initial clinical manifestation finding of the incoming abscesses in several children, delaying the diagnosis up to the moment when a neck mass, torticollis or nuchal pain appear [32].

Incidence of complications in pediatric DNIs is relatively low. Most cases recover uneventfully after proper antibiotic therapy and abscess drainage when needed [44]. Younger age, underlying chronic diseases and retropharyngeal space location are considered as risk factors for DNI complication development [45]. Upper airway obstruction has been described in case of large abscess or because of pharyngeal or laryngeal phlegmon or edema [46]. Mezenes et al. [47] reported the case of a 4-year-old boy with a PTA that developed dyspnea with stridor in the first day of conservative therapy. Early tonsillectomy was necessary to achieve complete resolution. Balasubramanian et al. [48] described a 4-month-old infant with a large, 5.3 × 8 cm^2^ RPA, which during a computed tomography (CT) scan showed significant airway narrowing. The patient underwent endoscopic assisted surgical drainage, was extubated 48 h after surgery and discharged after 1 week of intravenous antibiotic therapy. Moreover, Bernardini et al. [49] reported the case of a five-year-old girl with RPA, lymphadenopathy and narrowing of the pharynx, with bulging of the posterior wall, resulting in partial airway obstruction. No surgery was needed, as the patients recovered after adequate antibiotics therapy.

PPA can be associated with jugular vein thrombosis (Lemierre’s syndrome), venous septic embolus, disseminated intravascular coagulopathy, carotid artery pseudoaneurysm or rupture [50]. Nervous and bone structures at the cervical level are not spared and epidural abscess, atlanto-axial subluxation, cervical osteomyelitis, spinal cord abscess and meningitis have been described [50]. Dawes, in a population of 21 children with RPA (mean age 39 months), described two mediastinitis requiring prolonged admission to the intensive care unit and ventilatory support. Furthermore, Sankaraman et al. reported a 9-year-old girl with a frontal brain abscess secondary to an ipsilateral PTA [51]. Oleske et al. [52] reported the case of a 14-year-old boy with PTA that evolved into cervical phlegmon and lung abscess. He recovered completely after surgical drainage and was discharged home on antibiotics. Septic shock can occur. Pericleous et al. [53] reported the case of a 3-month-old girl that developed sepsis and desaturation. She required immediate intubation and admission to the intensive care unit (ICU) but recovered soon after the diagnosis of PTA was made, surgical drainage was performed, and broad-spectrum antibiotic treatment was started. Interestingly, cases of complicated DNIs have been described within the clinical picture of Kawasaki syndrome. Ravi et al. [54] presented the case of a seven-year-old male with neck pain and fever. Five days later, suspecting PTA, the boy underwent surgical drainage with evacuation of the purulent material and a broad-spectrum intravenous antibiotic therapy. Despite this therapy, the boy never fully recovered and two days after surgery, he developed new symptoms (i.e., bilateral conjunctival injection, diffuse macular rash, dry cracked lips, strawberry tongue, bilateral inguinal lymphadenopathy) [54]. Therefore, a diagnosis of Kawasaki syndrome was made, and intravenous immunoglobulin were infused with complete recovery of the patient. A similar case has been described by Isidori et al. [55].

## 4. Diagnosis

Clinical findings can suggest DNI. However, a definite diagnosis can be made only after the infection is exactly located and it has been defined if the infection has caused simple cellulitis or an abscess. Moreover, complications leading to life-threatening conditions for which an immediate resolutory approach is needed must be excluded. All these problems can be faced only with the use of imaging techniques. Definition of the disease etiology can significantly favor the right choice of antibiotic therapy. Pus collection from the site of infection, where possible, is strongly recommended. Finally, blood tests that measure the inflammatory response of the patient may contribute to confirm a diagnosis and monitor disease evolution.

### 4.1. Radiological Imaging

Lateral neck radiographs, several years ago used in the diagnostic work-up of DNIs [56], are much less used today, especially since it has been shown that the most modern methods of radiological investigation have much greater diagnostic sensitivity [57] and can give more reliable information to decide whether a surgical approach is needed [58]. Moreover, a chest X-ray remains important if complications such as mediastinitis, pneumomediastinum, lower airway foreign body or empyema are suspected.

Presently, CT is considered the method of choice for studying DNIs [59,60,61]. Contrast-enhanced CT (CECT) must be obtained as a non-contrast CT scan is significantly less accurate in the differentiation of cellulitis from abscess [62]. Compared to clinical examination, CECT has greater sensitivity (95% vs. 55%). However, specificity is lower (53% vs. 73%), mainly because detection of drainable abscesses smaller than 3.5 cm remains difficult. With small abscesses up to a 25% false positivity is reported [63]. Moreover, a CECT scan allows to accurately study the relationship between the abscess site and the neighboring structures, such as the major vessels of the neck, knowledge that is essential to perform drainage of an abscess, with significant mitigation of the surgical risk [62]. 

Intraoral or transcervical ultrasonography (US) can be the best solution for the evaluation of superficial lesions such as cervical adenitis and PTA and for percutaneous image-guided aspiration or drainage of pus. In differentiating abscesses from cellulitis, US is similarly sensitive and more specific than CECT. A study in children with lateral neck abscesses undergoing incision and drainage reported that the US positive predictive value was 96% compared to 88% of CECT whereas the negative predictive values were 16% for US and 6% for CECT [63,64,65]. Intraoral US seems equally effective than transcutaneous US. In a study, an intraoral US sensitivity of 95.2% was found, with a specificity of 78.5% and accuracy of 86.9%. On the contrary, transcutaneous US had 80% sensitivity, 92.8% specificity and 84.5% accuracy [66]. Unfortunately, together with some advantages, US has several limitations. It is a method of immediate use, allowing prompt choices for further diagnostic interventions and for the initiation of the most appropriate therapy. Moreover, it reduces utilization of a CECT scan, avoiding ionizing radiation to children. However, deeper neck spaces are rarely discernable by US, thereby limiting its diagnostic efficacy in many DNIs [67]. Use in children, especially if the oral probe is chosen, can be very difficult. Finally, US is a strictly operator-dependent method, thus the ability of the physician performing US is critical for the final diagnostic and therapeutic decisions.

Magnetic resonance imaging (MRI) provides superior soft-tissue characterization than both US and CT, leading to a more accurate differentiation of abscess from cellulitis or lymphadenomegaly in both children and adults [68]. A study has shown that despite the differences in the infection foci, emergency MRI in children had equal diagnostic accuracy than that in adults [69]. Moreover, MRI can be a useful and non-invasive method for diagnosing life-threating complications [70] and MRI findings can have high prognostic significance. Heikkinen et al. reported that the maximal abscess diameter and retropharyngeal edema evidenced by means of MRI were independent predictors of the need for intensive care unit (ICU) treatment whereas maximal abscess diameter and mediastinal edema were independent predictors of length of hospital stay [69]. Finally, it is associated with low radiation exposure [68,69,70]. Nevertheless, MRI is currently less often used in emergency settings as the first or only imaging modality, most likely due to its lack of availability, longer scanning time, reporting difficulty and higher cost. Moreover, in children, sedation issues can further limit its use [71,72,73].

### 4.2. Microbiology Tests

The identification of the pathogen is necessary to start targeted antibiotic treatment and to avoid treatment failure, longer duration of hospitalization and risk of life-threatening complication development. Obtaining the appropriate specimens from DNIs is crucial, as a variety of organisms can be isolated. Specimens can be collected at the time of surgical drainage or needle aspiration. Throat swabs or swabs obtained after drainage are inappropriate because they can be contaminated by oropharyngeal flora [73]. The methods for detection and quantification of microorganisms plays an important role. Specimens for culture should be transported by specific systems to facilitate the growth of both aerobic and anaerobic bacteria. In the laboratory, they should be inoculated and incubated into proper media to optimize the recovery of these organisms [29,73]. To improve detection of infecting pathogens, molecular methods can be used. They assure reliable responses in much faster times and have the advantage of detecting no-longer-viable bacteria, thus overcoming a relevant limit of the traditional culture. However, they require complex and expensive equipment, as well as experienced technicians. Finally, they may have some limitations when several bacteria are the cause of disease and the method does not take in account a priori all those in the exudate [74]. In some cases, generally the most severe, as they can associated with septicemia, a blood culture can be effective in the identification of the infecting pathogens. Unfortunately, this occurs rarely because of the empirical therapy frequently administered at home before hospitalization and blood sampling. In this case, Gram staining, acid-fast staining of the abscess content and throat swabs are useful to identify the pathogen [24].

### 4.3. Laboratory Tests

As DNIs are of bacterial origin, laboratory tests usually show neutrophilic leukocytosis, together with an increased serum concentration of C-reactive protein (CRP) and procalcitonin (PCT). Schraff et al., in a 10-year review on PTA in children, reported a mean value of 15.5 × 10^9^/L for leukocytes (range of 5.90–35.0), with a left shift in blood count (neutrophils 74% and lymphocytes 17%) [75]. Interestingly, in some cases, markers of Epstein–Barr virus infection can be detected [76], but it is not known whether this virus can be a trigger of a superimposed bacterial infection leading to DNI or the concomitant detection of these agents is a chance event.

## 5. Treatment

**Figure 3:**
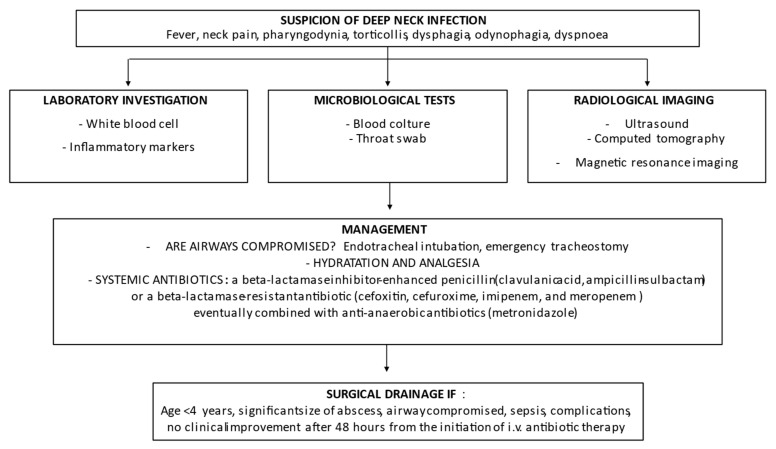
Flow-chart on management of deep neck infections in pediatric age.

Some aspects of management of pediatric DNIs remains debated. Evaluation and treatment of respiratory problems is the first step, to which all the experts agree. Debated is, on the contrary, the need for a surgical approach. Immediate surgical drainage has been the traditional mainstay of treatment for several years. Recently, a number of studies have shown that non-operative management can be effective in selected cases. Pooling the results of eight studies, it was calculated that the success rate of medical therapy in avoiding surgical drainage was 0.517 (95% confidence interval (CI): 0.335–0.700) [44]. Starting from the evidence that children ≥ 4 years and with abscess ≤ 25 mm were less likely to need surgical drainage, it has been suggested that older children with small abscess in stable conditions initially receive a course of high-dose intravenous antibiotics, are strictly monitored and undergo surgery only when medical treatment fails [77,78]. Regardless of age and abscess size, the presence of a compromised airway, septicemia or neurovascular complications suggest immediate surgical treatment [56]. However, some experts do not agree with this statement, stating that the evaluation of abscess site and size can be difficult even when advanced diagnostic measures are used [79]. On the other hand, further factors, together with age, size of the abscess and presence of complications, can condition management. Comorbidities, difficulties in performing diagnostic procedures and lack of potentially effective antibiotics in low socioeconomic environments can favor surgery. This explains why in an evidenced-base review of DNIs in children, it was suggested that the therapeutic approach should be targeted toward the individual patient [58]. Figure 3 summarizes the flow-chart that can be followed in clinical practice. It must be highlighted that, in children under 18 months of age with a previous diagnosis of upper respiratory tract infection, the persistence of fever and the worsening of the general conditions must lead one to think about the possible presence of a DNI and to establish all the diagnostic measures already put in place in older children with early and reliable signs and symptoms of these diseases.

### 5.1. Medical Treatment

Regardless of the surgical treatment, antibiotics are critical for pediatric DNI prognosis. Considering the microbiological characteristics of DNIs, parenteral administration of broad-spectrum antibiotic therapy covering both aerobic and anaerobic pathogens is strongly recommended. Adjustments may be needed when the etiology of disease is precisely defined through culture or molecular biology methods [78]. Moreover, when a patient improves, parenteral therapy may be switched to oral therapy with the same drug(s) if available or with drugs with a similar spectrum of activity. Generally, a beta-lactamase inhibitor-enhanced penicillin (amoxicillin-clavulanate, ampicillin-sulbactam) or a beta-lactamase-resistant antibiotic (cefoxitin, imipenem, meropenem), eventually combined with an anti-anaerobic drug (clindamycin or metronidazole), is recommended [48]. Patients with previous anaphylactic reactions to penicillin may be treated with clindamycin [80,81]. However, particular attention must be paid to the problem of antibiotic resistance. Choice of the initial antibiotic therapy must carefully consider the rates of antibiotic resistance of the most common pathogens of pediatric DNIs in the geographic area where the patients is treated.

The use of corticosteroids in association with antibiotics in patients with DNIs is debated, although these anti-inflammatory drugs are frequently associated with the antibiotic administration in many pediatric studies. With some exceptions [82], these drugs do not significantly modify the course of a bacterial disease when given with an effective antibiotic therapy. Regarding DNIs, some but not all the studies carried out in adults have found that pain scores, mouth opening, time to painless oral intake and duration of hospitalization were significantly improved in patients receiving corticosteroids compared to those given placebo [83]. In children, studies are too few to allow definitive conclusions to be drawn. In the most recent reports, dexamethasone was given to 35% of 153 patients. The treated children had a significant decreased rate of surgical drainage (36% and 53%, respectively; *p* = 0.043). Moreover, the treated patients had a shorter duration of hospital stay, but the difference was not statistically significant (2.9 days and 3.8 days; *p* = 0.09) [84].

### 5.2. Surgical Approach

In front of a PTA, three options are possible: needle aspiration, incision and drainage, and abscess tonsillectomy. The choice of the treatment depends on the healthcare personnel’s skills and experience, patient cooperation, cost and whether the patient has indications for tonsillectomy. Incision and drainage are painful procedures that can cause considerable bleeding. Needle aspiration is supposed to be significantly safer and better tolerated. Despite there not being enough evidence for drawing firm conclusions in children, studies in adults seem to indicate that this procedure is less painful than incisions and drainage [85]. Tonsillectomy in PTA is controversial: it should be considered in patients with recurrent tonsillitis, obstructive sleep apnea or in case of the failure of other techniques [86].

For PPA and RPA, an oral approach is suggested when possible [56]. However, PPAs (that are only partially seen in the pharynx) and complicated RPAs must be treated by an external cervical approach as the intraoral approach can be dangerous or would allow full drainage [58]. However, surgery should be preceded by a careful evaluation of the patency of the airways. In some cases, risk of obstruction is very high. Typically, endotracheal intubation solves this problem, but in some cases this procedure can be difficult to perform for edema of the larynx and abscess protrusion or can be dangerous because it can increase the edema or cause the rupture of the abscess with the aspiration of pus into the airways. An emergency tracheotomy can be required, and this explains why some experts suggest that this must be anticipated in all cases of severe DNI. Monitoring of the respiratory tract should continue for at least 48 h after surgical intervention because of the potential for increasing edema in the postoperative period [87].

## 6. Conclusions

Despite being relatively uncommon, pediatric DNIs deserve particular attention as they can have a very severe course, lead to admission to an intensive care unit and, although very rarely, death. Risk of respiratory failure or severe complications involving muscles, vessels and nerves of the neck can be avoided only with an early diagnosis and a prompt antibiotic and surgical approach. However, the diagnostic-therapeutic procedure to be followed in the individual patient is not universally shared because it is not established which is the most valid radiological approach and which are the criteria to be followed for the differentiation of cases to be treated only with antibiotics and those for which surgery is mandatory. Further studies are needed to define the standardized guidelines for ensuring the best possible care for children with DNIs, especially in this era of increased antimicrobial resistance.
